# Accuracy of intraoral scanners for full-arch implant scanning in the Maxilla using horizontal scanbody

**DOI:** 10.1371/journal.pone.0332174

**Published:** 2025-12-05

**Authors:** Abidiel Silva Guimarães, Thais Silva Mendonça, Paulo Sergio Borella, Gus Khalil, Junying Li, Sandra Al-Tarawneh, Lawrence Gonzaga Lopes, Gustavo Mendonça

**Affiliations:** 1 Department of Oral Rehabilitation and Prevention, School of Dentistry, Federal University of Goiás, Goiania, Goiás, Brazil; 2 Department of Prosthodontics, School of Dentistry, Virginia Commonwealth University, Richmond, Virginia, United States of America; 3 AOX Digital Designs, Orlando, Florida, United States of America; 4 Department of Biological and Materials Sciences & Prosthodontics, School of Dentistry, University of Michigan, Ann Arbor, Michigan, United States of America; Universiti Sains Malaysia, MALAYSIA

## Abstract

This laboratory study evaluated the accuracy of six intraoral scanner models (Primescan; Trios 4; Trios 5; Medit i700; i900; Itero Lumina) compared to a desktop scanner (E4) used as a control. Additionally, the influence of the scan body design, horizontal (SBH) and short cylindrical (SBC), was investigated through two different software. A master model containing six implants was used, and the scanning workflow was standardized and repeated ten times (N = 10). The data were analyzed in terms of trueness and precision using two distinct types of analysis: one performed in Blender software (Stichting Blender Foundation) and the other in Evalumap software (Claronav). The lower the number the better precision and accuracy were noted. The position deviation (3D_dev) and angular deviation (Ang_dev) were evaluated. Statistical analyses were conducted using the Kruskal-Wallis test, Bonferroni correction, and multiple comparisons. Trueness (n = 1,437) showed a significant effect on 3D_dev (p < 0.001) and Ang_dev (p < 0.001), as well as for precision (n = 6,478) in 3D_dev (p < 0.001) and Ang_dev (p < 0.001). The lowest median values for trueness in 3D_dev were observed for the i900L (35.25) and in Ang_dev for Primescan (0.35), whereas the lowest precision values for 3D_dev were found for the iTero Lumina (18.81μm) and Ang_dev in i900L (0.19 μm). Regarding the scan body type, a significant difference was observed between SBH and SBC for both trueness and precision, with SBH presenting lower angular deviation values and SBC exhibiting lower 3D position deviations. Multiple comparisons revealed significant differences for the E4 scanner across all analyses. The choice of scanner impacts both the trueness and precision of the generated models, as does the scan body type and the software.

## Clinical implications

Accurate full-arch implant scans are critical to achieving passive fit and minimizing mechanical complications. This study shows that scanner and scan body selection significantly affect digital impression accuracy. Clinicians should prioritize systems that minimize positional and angular deviations to improve prosthesis fit. Tools like Evalumap can assist in chairside scan evaluation, reducing errors and enhancing efficiency in the digital workflow.

## Introduction

The use of intraoral scans in dentistry is a well-established practice widely applied for various clinical and laboratory purposes [1–3]. Continuous advancements in intraoral scanners, as well as associated hardware and software technologies, have enabled the development of increasingly predictable, precise, and comfortable therapeutic techniques for patients [4,5]. However, significant challenges persist, particularly in making digital impressions for full-arch implant cases in the maxilla, where conventional impressions often yield more accurate results [6]. While obtaining intraoral implant scans with sufficient fidelity and accuracy to fabricate prostheses within a digital workflow remains technically challenging and dependent on the condition being scanned [7], recent studies suggest that digital workflows may outperform analog methods for implant position transference [8,9].

Inaccurate models for implant-supported restorations can result in marginal misfits, non-passive structures, excessive adjustments, occlusal discrepancies affecting functionality, and biomechanical complications such as mechanical overload and imbalanced forces on implants. These issues increase the risk of screw loosening, fractures, and even implant failure, potentially compromising treatment longevity [10–13]. Furthermore, these complications can negatively impact the patient experience by causing discomfort, aesthetic and functional dissatisfaction, increased treatment costs, and more dental visits. Shorter and more precise treatments with immediate results tend to enhance patient satisfaction [14].

Intraoral scanning of implants presents various technical and clinical challenges that can compromise the accuracy and predictability of restorations. Key difficulties include implant position, anatomical interferences, scan body geometry, patient movement, cumulative processing errors, implant distances, operator skill, and scanner capability [9,15–18]. Scanner accuracy can be affected by intrinsic limitations such as capture technology, resolution capability, and processing software [19,20].

Scan bodies serve as geometric references, transferring the implant’s three-dimensional (3D) position to planning software. They must be designed to facilitate capture by intraoral scanners, featuring flat surfaces and well-defined geometric markers, opaque material, stability, and appropriate height to ensure full tissue visualization. They should not interfere with scanner access in the oral cavity and should minimize alignment errors with the digital counterpart [16,20,21]. A prototype scan body with a 16 mm horizontal bar and a hemisphere at its end horizontal scan body (SBH) has been developed to provide a visual connection without true material union. This design partially aligns with findings by Ashraf et al [22], who observed that true union positively affected scanning accuracy for full-arch cases. In a recent accuracy study, Meneghetti et al. [23] evaluated the accuracy of producing 3D models for maxillae with six implants. They compared various scan bodies and found that a short cylindrical scan body with a diameter of 7 mm (SBC) yielded the best results. Additionally, they observed a difference in accuracy among the different scanners tested.

Accuracy assessment involves analyzing two main components: trueness and precision. Trueness refers to the system’s ability to faithfully capture the object’s actual geometry, measuring how closely the digital model matches the physical object. Precision, on the other hand, evaluates the consistency or reproducibility of scans, measuring the proximity of multiple scans of the same object. Accuracy evaluation requires a standard reference model as a comparison baseline. Trueness is determined by comparing generated models with the reference, while precision is assessed by analyzing variations among models from the same set. Specialized metrology software, including tools with best-fit alignment capabilities, is often used to align and compare models, ensuring detailed and precise analysis [24].

Given these challenges, further research and technological development are essential to ensure the reliability and feasibility of these processes in clinical and laboratory contexts. Thus, the research question arises: Can different intraoral scanners produce distinct 3D images regarding the three-dimensional position of implants using two types of scan bodies?

This study aimed to assess the accuracy of six intraoral scanners and one reference desktop scanner in capturing digital impressions of implants in the maxilla using both short and horizontal scan bodies, under two distinct software programs. The evaluation was performed using two computational systems, focusing on deviations in the 3D position, angulation, and implant position within the arch. The null hypothesis is that there would be no differences in the accuracy among the tested scanners, the use of horizontal scan body, and implant position within the arch.

## Materials and methods

**Fig 1:**
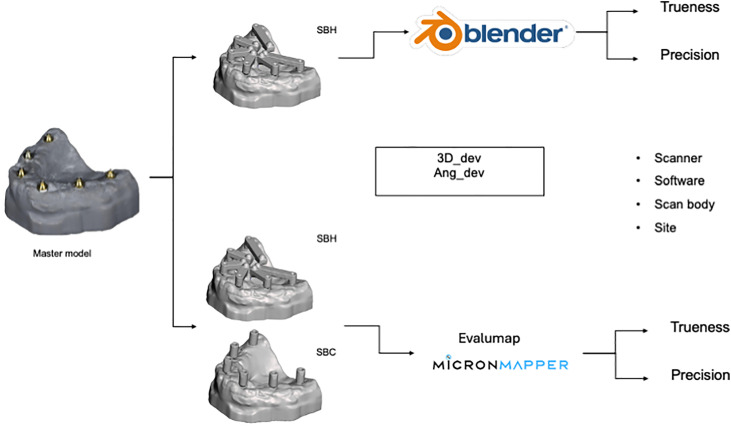
Flow of research method.

To evaluate the accuracy of the scanners, two methods of analysis were established using different software. The first method involved using Blender (Stichting Blender Foundation, Amsterdam, Netherlands), between specific points (apical, platform, and coronal) on the scan bodies. The second analysis was conducted using Evalumap (Claronav, Toronto, Canada), where the implant position files were converted via a script (S1) in Blender. Both methods measured the distance deviation (3D_dev) and angular deviation (Ang_dev) from the reference scan, providing the accuracy values. The analyses in Blender were performed using SBH, while in Evalumap, both SBH and SBC were evaluated (Fig 1).

### Reference master model

A master 3D-printed model of an edentulous maxilla with six implants (in the regions of teeth #3, #5, #7, #10, #12, and #14) with abutments installed (4.1 mm x 10 mm, SIN Implant System, Brazil) (Fig 1) was used. A digital impression of the master model was made using a bench scanner (E4, 3Shape A/S, Copenhagen, Denmark), which served as the reference for the alignment of all subsequent objects [25]. A total of ten scans were performed with each scanner model (n = 10).

### Model scanning process

Digital impressions were performed using seven different scanners to assess the accuracy of different intraoral scanners on full-arch models with six implants. The same scanning protocol was performed for all scanners using both the SBH and SBC. All digital impressions were performed by an experienced, trained, and calibrated operator. The scanning was continuous without interruptions, starting with the occlusal/buccal surface of implant SB #3 to #14, returning through the lingual to #3 and complementing each SB with circles with a 45-degree inclination, as applied in Menegethi et all 2023 [23]. Seven scanners were selected for this study: E4 (3Shape A/S, Copenhagen, Denmark), Trios 4 (3Shape, Copenhagen, Denmark), Trios 5 (3Shape, Copenhagen, Denmark), i700, i900 with the large tip, i900 with the Mediun tip, Primescan, Itero, and the E4 itself. Table 1 specifies the scanners used.

**Table 1:** Descriptions of scanners.

Nome	Fabricante	Acquisition	Output files
E4	3Shape A/S. Copenhagen, Denmark	Structured Light Scanning.	Dcm (propriety format), with the possibility to export.stl files (open formats with Trios on Dental Desktop).
Trios 4	3Shape, Copenhagen, Denmark	Confocal Microscopy and Ultrafast Optical Scanning	Dcm (propriety format), with the possibility to export.stl files (open formats with Trios on Dental Desktop).
Trios 5	3Shape, Copenhagen, Denmark	Confocal Microscopy and Ultrafast Optical Scanning	Dcm (propriety format), with the possibility to export.stl files (open formats with Trios on Dental Desktop).
Medit i700	Medit, Seoul, South korea	3D in Motion Video Technology	Possibility to export.stl files
Medit i900	Medit, Seoul, South korea	3D in Motion Video Technology	Possibility to export.stl files
Primescan	Dentsply Sirona, York, PA USA	High-resolution Sensors and Shortwave Light with Optical HighFrequency Contrast Analysis for Dynamic Deep Scan (20 mm)	Dxd (property format) with the possibility to export.stl files (open format) with Cerec Connect.
iTero Lumina	iTero, Align Technology	Multi-Direct Capture Technology Capture distance of up to 25 mm	Dcm (propriety format), with the possibility to export.stl files, and can be exported for integration with cone beam CT data (open formats with Trios on Dental Desktop).

### Evaluation of implant position

#### Blender.

**Fig 2:**
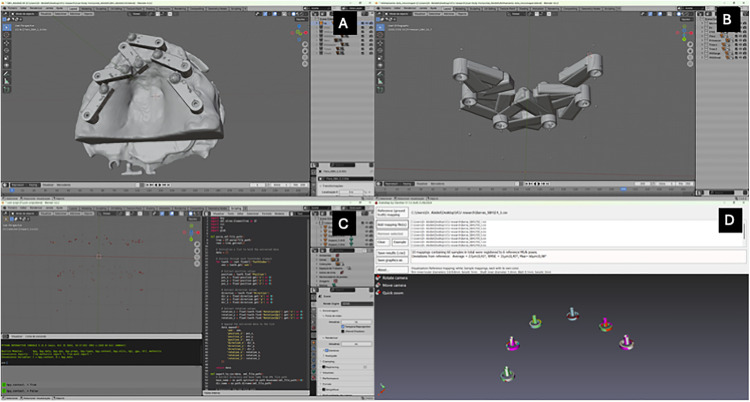
Blender and Evalumap analysis. (A) Initial positioning of the aligned models in the Blender software; (B) After the removal of the models, the scan bodies with the dots remained; (C) After the removal of the scan bodies and introduction of the scrip to measure the distance between the dots; (D) Analysis of the implant positions in the Evalumap software.

The scans generated models in their respective native software formats or in.stl format, which were then exported to a design software (3Shape Dental Manager Premium 2023 software, 3Shape A/S, Copenhagen, Denmark). Each digital model file was subsequently aligned with the master model (Fig 2 A and B). To record the 3D position of the implants, a digital scan body.stl file was created, featuring five spheres distributed in the following positions: coronal, multiunit platform, lateral to the platform, implant platform, and apical of the implants (Fig 2 C). This setup aimed to capture the 3D position of each implant in the xyz axes, as well as its recorded angulation.

For scan body alignment, a three-point alignment tool was used, and the aligned SBH was saved as an.stl file. The files were then exported to an open-source software (Blender version 2.93) in organized and named collections for each scanner model. These collections were processed through object separation by loose parts, and a Python script was used to rename the objects. The separation and renaming of objects generated files that could be analyzed via a Python script to assess deviations in position and angulation between the digitized reference master model and their corresponding intra-group homologs. Finally, another Python script was developed to export two.csv files corresponding to Precision and Trueness of the collected data, according to what was executed by Meneghetti et al [23].

Accuracy evaluation was performed by assessing both precision and trueness, considering both the distance from the platform and the implant angulation. The precision data for distance and angle referred to the proximity of repeated measurements to one another, indicating the dispersion of results around a mean or central value. Trueness evaluation was based on the comparison of measurements to the reference value of the master model.

### Evalumap analysis

The strategy employed involved using design software (3Shape Dental Manager Premium 2023, 3Shape A/S, Copenhagen, Denmark) to generate numerical data, precisely the coordinates of implant positions. This software was used to produce protocol bars, which facilitated the extraction of 3D positional information for the implants (Fig 2 D). The software automatically generated text files containing each implant’s 3D positions and angulations. These files were stored within the case folder as part of the software’s output.

To further process the data, a Python script was developed to convert the text file format into a.csv file. This conversion aligned with the positional data of each implant as obtained from the scan and documented during the protocol bar creation process. Each.csv file was opened in the software (Evalumap Claronav) used to check the fitting of restorations, where comparisons of 3D distance deviation (3D_dev) and angular deviation (Ang_dev) between the reference model and each scanned model were conducted. Trueness comparisons were made between the reference model and the models from each group, generating 60 comparisons per model. For Precision, all models within each group were compared to one another, generating a total of 270 comparisons. The lower the number the better precision and accuracy were noted.

### Statistical analysis

The obtained data were tabulated in an Excel spreadsheet and subjected to statistical analysis using a software package (SPSS 29, IBM). Initial comparisons presented precision and trueness data for 3D_dev and Ang_dev of the scanner for both software (Blender and Evalumap) using two scan bodies (SBH and SBC). Pairwise comparisons showed significant differences for precision, trueness, 3D_dev, and Ang_dev inside groups.

The precision and trueness data were initially assessed for normality using Shapiro-Wilk test, which revealed a non-normal distribution. Subsequently, the Kruskal-Wallis test was applied to compare scanners or sites, followed by Bonferroni correction and multiple comparisons. For comparisons between the Blender and Evalumap software, as well as between the SBH and SBC scan bodies, the Mann-Whitney test was used. All tests were conducted with an alpha of 0.05 and a power of 0.80.

## Results

The descriptive analysis initially presented the median, minimum, and maximum values for each scanner in terms of 3D_dev and Ang_dev. The E4 scanner showed the highest precision, followed by the iTero, which showed the highest precision among the intraoral scanners, while the i900M scanner presented the lowest precision (Table 2).

**Table 2:** Descriptive analysis of scanners.

		Median		Minimun		Maximun	
Scanner		Trueness	Precision	Trueness	Precision	Trueness	Precision
E4	3D_dev	11.48	11.55	0.00	0.50	160.40	186.80
	Ang_dev	0.13	0.09	0.00	0.00	1.96	0.65
i700	3D_dev	40.40	36.10	1.70	0.50	131.60	188.70
	Ang_dev	0.38	0.24	0.30	0.00	2.08	1.03
i900L	3D_dev	35.25	28.18	1.40	0.70	104.90	87.60
	Ang_dev	0.37	0.15	0.01	0.00	1.72	0.93
i900M	3D_dev	45.06	53.23	0.90	0.40	460.85	451.65
	Ang_dev	0.40	0.31	0.02	0.00	1.70	2.10
iTero	3D_dev	37.50	18.81	8.30	1.58	160.80	61.77
	Ang_dev	0.37	0.19	0.04	0.01	1.57	0.89
Primescan	3D_dev	43.65	37.39	2.20	0.00	141.65	155.70
	Ang_dev	0.35	0.24	0.02	0.00	1.73	2.18
Trios4	3D_dev	46.20	41.71	3.30	0.30	377.09	369.25
	Ang_dev	0.34	0.23	0.01	0.00	1.82	1.76
Trios5	3D_dev	61.19	48.67	3.20	0.00	148.00	228.80
	Ang_dev	0.37	0.24	0.04	0.00	2.16	0.89

* Angular deviation (Ang_dev)

### Scanners comparisons

**Fig 3:**
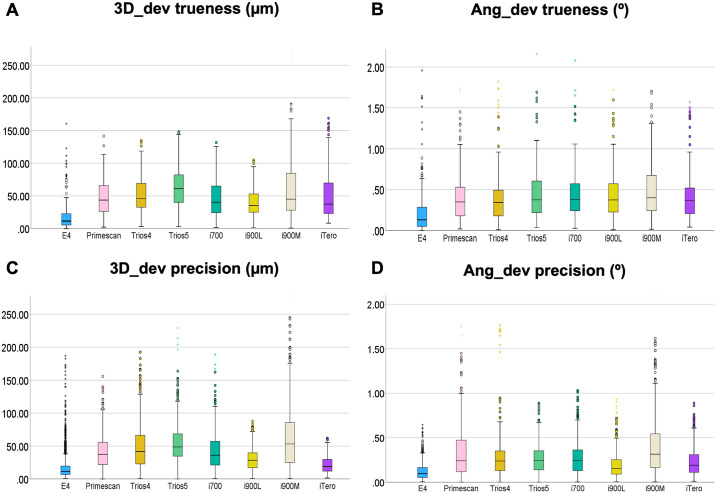
3D and angular accuracy. Independent-Samples analysis Kruskal-Wallis for scanners.

**Fig 4:**
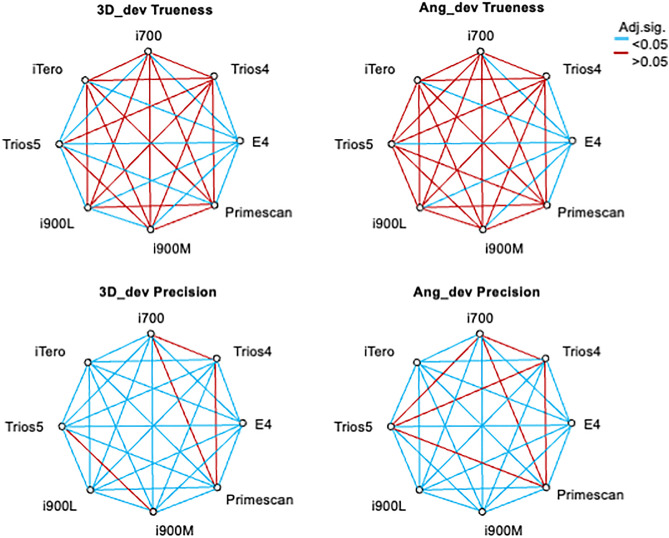
3D and angular accuracy. Independent-Samples analysis Kruskal-Wallis for scanner interactions in Bonferroni`s correction.

Subsequently, the Kruskal-Wallis test for independent samples was performed, followed by Bonferroni correction and multiple comparisons. For trueness (n = 1,437), the analysis showed a significant group effect on 3D_dev trueness [x²(7)=254.51; p < 0.001] and Ang_dev trueness [x²(7)=132.75; p < 0.001], as well as on precision (n = 6,478) for 3D_dev [x²(7)=1,419.81; p < 0.001] and Ang_dev [x²(7)=819.76; p < 0.001]. Multiple comparisons revealed significant differences for the E4 scanner in all comparisons (Figs 3 and 4).

### Software and scan body comparisons

**Fig 5:**
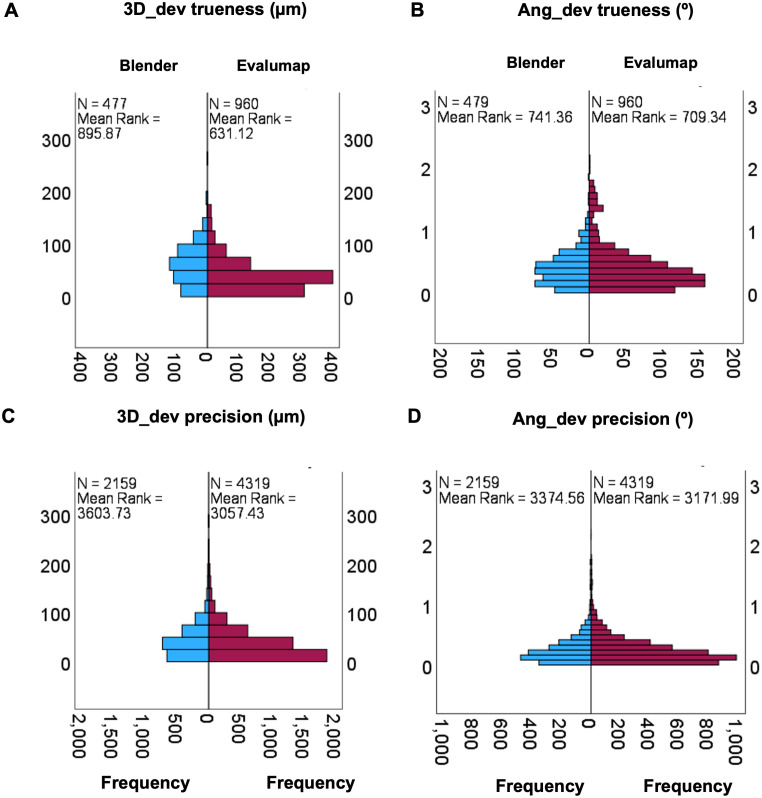
Independent-Samples analysis Mann-Witney U for software value trueness and precision. A – Comparison in 3D_dev trueness; B – Ang_dev trueness; C – 3D_dev precision; D – Ang_dev precison.

The results were analyzed using the Mann-Whitney test for independent samples, considering differences in trueness (n = 1,437) and precision (n = 6,478). For trueness, 3D_dev showed a statistically significant difference (U = 144,595, p < 0.001) (Fig 5 A), while Ang_dev did not reach significance (U = 219,638, p = 0.168) (Fig 5 B). For precision, both 3D_dev (U = 3,178,121, p < 0.001) and Ang_dev (U = 4,370,770, p < 0.001) (Fig 5 C and D) showed significant differences. Although the sample sizes were numerically different, the Mann-Whitney test is robust in assessing distributions. The observed significance reflects real differences in mean ranks, especially in the case of 3D_dev, where the distributions showed less overlap. On the other hand, the lack of significance in Ang_dev for trueness indicates greater similarity between the distributions.

**Fig 6:**
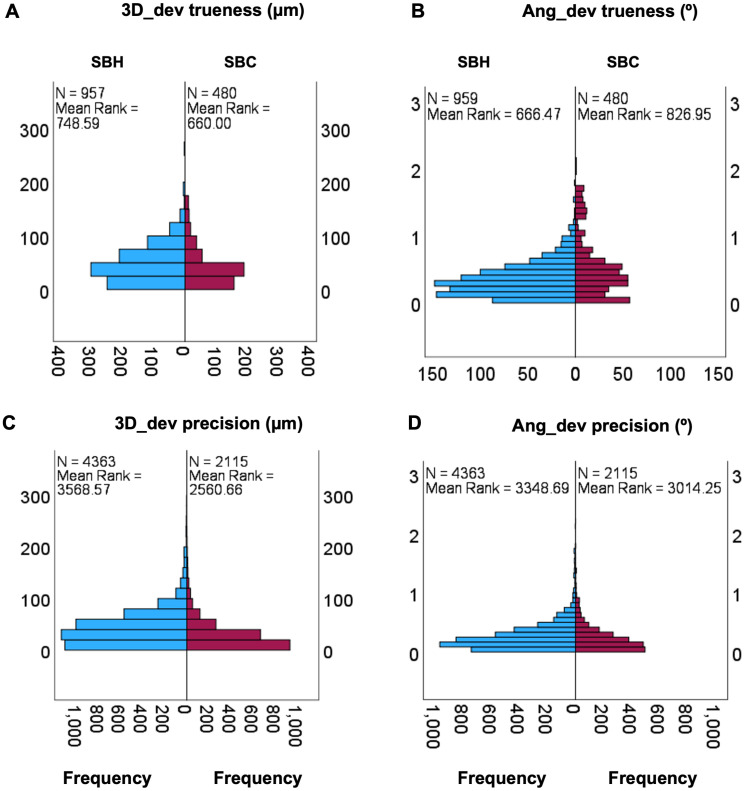
Independent-Samples analysis Mann-Witney U for scan body value trueness and precision. SBH * SBC. A –3D_dev trueness; B – Ang_dev trueness; C – 3D_dev precision; D – Ang_dev precison.

The Mann-Whitney test for independent samples was also applied to the scan body data to evaluate differences in trueness (n = 1,437) and precision (n = 6,478). For trueness, 3D_dev showed a statistically significant difference (U = 201,359.00; p < 0.001), as did Ang_dev (U = 281,496.00; p < 0.001) (Fig 6 A and B). Similarly, for precision, both 3D_dev (U = 4,137,464.00; p < 0.001) and Ang_dev (U = 4,137,464.00; p < 0.001) presented significant differences (Fig 6 C and D). The visual distribution of the data highlights the observed differences between the groups. Despite the numerical disparity in sample sizes, the Mann-Whitney test remains robust, reflecting substantial differences in mean ranks across all comparisons.

**Fig 7:**
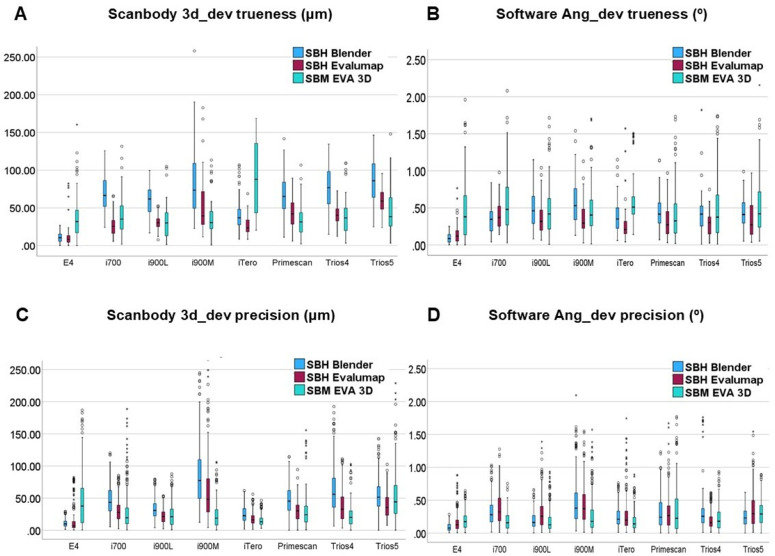
Boxplot pairwise Scan body to software and scanner. Boxplot graphic showing difference between scan bodies inside each scanner for 3D deviation trueness and precision.

**Fig 8:**
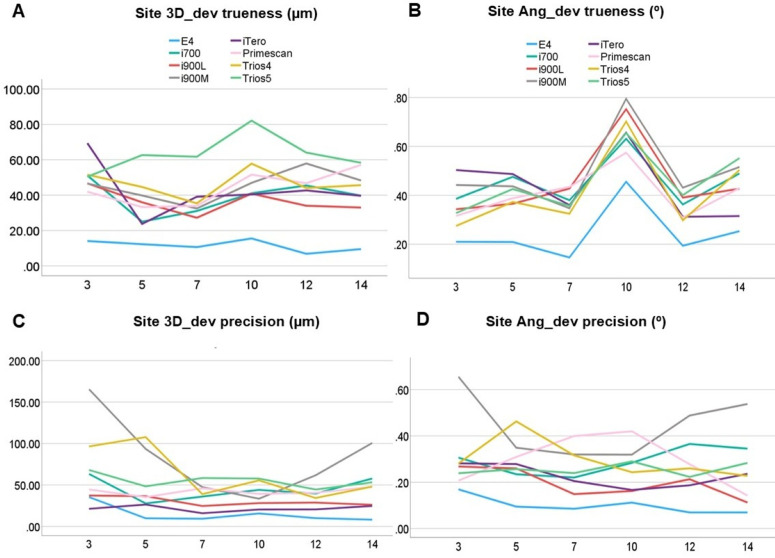
Pairwise site to 3D_dev and Ang_dev for scanner. Graphic showing difference between each scanner for 3D deviation trueness and precision.

Descriptive analyses of the medians in trueness and precision of the scan bodies SBH and SBC and of the implant site with the values produced within the Evalumap software and the SBH scam body in the Blender and Evalumap software, Figs 7 and 8, were performed.

### Site comparisons

The Kruskal-Wallis test revealed significant group effects across all evaluated parameters. For precision, there were significant differences among groups in 3D_dev [χ²(5)=154.59; p < 0.001] and Ang_dev [χ²(5)=20.12; p = 0.001]. Similarly, for trueness, significant differences were observed in 3D_dev [χ²(5)=32.26; p < 0.001] and Ang_dev [χ²(5)=5277; p < 0.001]. The graphical representation (Fig 8) illustrates the variability across groups, with notable peaks and trends in precision and trueness values for specific scanners. The results indicate distinct performance patterns among the devices, with some demonstrating higher precision and trueness under specific conditions. These findings underscore the variability in scanner performance.

## Discussion

The master model was scanned using seven different intraoral scanner models and one desktop scanner. This enabled a variety of analyses to be conducted using both Blender and Evalumap software. Following the research objectives, it was observed that the different scanner models produced statistically significant differences in both trueness and precision, leading to the rejection of the null hypothesis. Regarding the scan body models, there were also statistically significant differences: the SBC yielded better results for 3D deviation (3D_Dev), while SBH showed better results for angular deviation (Ang_Dev), which also led to the rejection of the null hypothesis. Similarly, the implant position showed statistically significant differences, prompting the rejection of the null hypothesis in this context as well.

For metric analysis, the Blender software provided dimensional property assessments based on methodologies described by Chen et al [26] and Meneghetti et al. [23]. The analyses involved programming the software with specific scripts to follow a meticulous workflow that ensured accurate measurements. Meanwhile, the Evalumap software used Blender not for measurements but to generate a.csv spreadsheet derived from a 3Shape-generated file from the 3D object. This file recorded the implant’s 3D position once the bar was created. This method required developing a custom Python script for Blender 4.1, a novel approach introduced in this study by the research team.

The use of two software programs for analysis offers the advantage of promoting new research methodologies, facilitating comparisons of results, enhancing the comprehension of implant position analysis techniques, and introducing a novel analytical model. Evalumap was developed to be used alongside the photogrammetry equipment (Micrommapper), designed to measure discrepancies between implant and prosthesis positions, making it a promising research tool. These software solutions were necessary to isolate implant data positions from soft tissues, as other reference standard methodologies would not reveal direction or angular deviation [24]. Fig 6 illustrates the differences observed across the software and scan body used models.

The scanners exhibited varying performance compared to the E4 desktop scanner, with all showing statistically significant differences. Notably, the desktop scanner consistently delivered the lowest deviation values, aligning with prior studies [25]. Comparisons among intraoral scanners revealed mixed results: some differences were significant, while others were not (Fig 2). For instance, the i900L scanner demonstrated the best median values for trueness, while the iTero Lumina excelled in precision.

The geometry of the SBC presented advantages by not affecting adjacent soft tissue scanning and achieving higher accuracy in 3D_Dev, consistent with previous studies [22,23]. Conversely, the SBH yielded the lowest Ang_Dev values, corroborating Ashraf et al. [22], who observed reduced angular deviation with visual connection artifacts between implants [22].

The implant site position also significantly influenced results. For trueness, both 3D_Dev and Ang_Dev showed higher values for implant #10. In terms of precision, 3D_Dev values were highest at the extremity implants, consistent with earlier findings that implant/scan body positions affect positional deviation [20,27,28]. Precision in Ang_Dev, however, showed uniformity (Fig 7). Factors such as implant position, scanning method, and scan body model significantly impact results [18,20–23,27–29].

While the practical differences may seem small, they can be clinically significant, particularly in ensuring prosthetic passivity. The material used, whether zirconia, titanium, or printed resin, can result in vastly different outcomes. However, the study did not disqualify any scanner, as all deviations were below 62 µm, unlike earlier studies that ruled out certain scanners for full-arch fixed prosthetic models [20].

Zirconia requires high accuracy due to its biomechanical properties, such as hardness and elastic modulus, necessitating corrective methods like cementation links [30,31]. For printed resins used as provisional prostheses, links may or may not be necessary. Additionally, a randomized clinical trial by Cappare et al. (2019). found no significant differences between digital workflows with milled titanium bars and conventional workflows [32].

Combining highly accurate scanners with well-designed scan bodies is critical for the success of implant-supported prosthetic rehabilitation in a digital workflow. The scan body’s geometry must be compatible with the scanner’s technology, ensuring faithful capture of its position and orientation on the implant [33]. In this study, a single trained operator performed all scans, following a consistent scanning protocol detailed in prior research [23]. While some studies suggest implant platform type has no impact [16,34]. a clinical verification step before final prosthesis production, such as using passivity test indexes, can help minimize negative impacts on digital model creation [35].

This study has limitations that should be considered. Multiple mesh alignments were required for the Blender group, potentially introducing variability into the results. Being an in vitro study, the findings may not fully replicate clinical conditions or challenges encountered in practice. Additionally, restorations were not fabricated to verify their clinical suitability, leaving the practical implications of the deviations observed untested. The Evalumap software, while effective, is bundled with a photogrammetry scanner, limiting its accessibility to a broader audience. Ongoing research and advancements in technology are crucial to overcoming these limitations, enhancing predictability and efficiency in digital workflow-based treatments, and improving clinical outcomes.

## Conclusion

Within the limitations of this in vitro study, it can be concluded that:

The i900L scanner demonstrated the highest trueness for 3D_Dev, while the Primescan had the highest trueness for Ang_Dev. For precision, the iTero Lumina showed the best results in 3D_Dev, and the i900L in Ang_Dev.

The horizontal scan body achieved the better Ang_Dev values, whereas the cylindrical scan body yielded the worse values for 3D_Dev.

The choice of scanner and scan body significantly affects both the trueness and precision of the digital impressions.

The evaluation process using the Evalumap software was found to be simpler and more practical. It demonstrated sufficient efficiency to assist in future research and chairside evaluations.

The implant position within the arch influences both trueness and precision.

## Supporting information

S1 Script Scripts used to extract information from files. (PDF)
