# Supplementary material for: Accuracy of intraoral scanners for full-arch implant scanning in the Maxilla using horizontal scanbody
Source: PLoS One. 2025 Dec 5;20(12):e0332174. doi: 10.1371/journal.pone.0332174 (PMC12680154; doi:10.1371/journal.pone.0332174)
Supplement: S1 Script — (PDF) [file pone.0332174.s001.pdf]

XML >> CVS file

```
import bpy
import xml.etree.ElementTree as ET
import csv
import os
import glob

def parse_xml(file_path):
    tree = ET.parse(file_path)
    root = tree.getroot()

    # Initialize a list to hold the extracted data
    data = []

    # Iterate through each ToothIndex element
    for tooth in root.findall('ToothIndex'):
        unn = tooth.get('unn')

        # Extract position values
        position = tooth.find('Position')
        pos_x = float(position.get('x') or 0)
        pos_y = float(position.get('y') or 0)
        pos_z = float(position.get('z') or 0)

        # Extract direction values
        direction = tooth.find('Direction')
```

```
        dir_x = float(direction.get('x') or 0)
        dir_y = float(direction.get('y') or 0)
        dir_z = float(direction.get('z') or 0)

        # Extract rotation values
        rotation_x = float(tooth.find('Rotation[@x]').get('x') or 0)
        rotation_y = float(tooth.find('Rotation[@y]').get('y') or 0)
        rotation_z = float(tooth.find('Rotation[@z]').get('z') or 0)

        # Append the extracted data to the list
        data.append({
            'unn': unn,
            'position_x': pos_x,
            'position_y': pos_y,
            'position_z': pos_z,
            'direction_x': dir_x,
            'direction_y': dir_y,
            'direction_z': dir_z,
            'rotation_x': rotation_x,
            'rotation_y': rotation_y,
            'rotation_z': rotation_z
        })

    return data

def export_to_csv(data, xml_file_path):
```

```
    # Extract directory and base name from XML file path
    base_name = os.path.splitext(os.path.basename(xml_file_path))[0]
    dir_name = os.path.dirname(xml_file_path)

    # Construct the CSV file path
    csv_file_path = os.path.join(dir_name, f"{base_name}.csv")

    # Write the data to the CSV file without headers
    keys = data[0].keys() if data else []
    with open(csv_file_path, 'w', newline='') as csvfile:
        writer = csv.DictWriter(csvfile, fieldnames=keys)
        writer.writerows(data)
    print(f"CSV file exported to: {csv_file_path}")

def process_xml_files(directory_path):
    xml_files = glob.glob(os.path.join(directory_path, "*.xml"))

    for file_path in xml_files:
        base_name = os.path.splitext(os.path.basename(file_path))[0]
        print(f"Processing file: {file_path}")

        # Create a new collection for this XML file
        collection = bpy.data.collections.new(base_name)
        bpy.context.scene.collection.children.link(collection)

        extracted_data = parse_xml(file_path)
```

```python
export_to_csv(extracted_data, file_path)


for item in extracted_data:
    pos_x = item['position_x']
    pos_y = item['position_y']
    pos_z = item['position_z']
    dir_x = item['direction_x']
    dir_y = item['direction_y']
    dir_z = item['direction_z']
    rot_x = item['rotation_x']
    rot_y = item['rotation_y']
    rot_z = item['rotation_z']
    unn = item['unn']

    # Create a new mesh (sphere)
    bpy.ops.mesh.primitive_uv_sphere_add(radius=0.25, location=(pos_x, pos_y, pos_z))
    sphere = bpy.context.object

    # Apply rotation to the sphere
    sphere.rotation_euler = (rot_x, rot_y, rot_z)

    # Additional settings for the object
    bpy.context.object.rotation_mode = 'XYZ'
    bpy.context.object.up_axis = 'Y'
    bpy.context.object.track_axis = 'POS_Z'
    bpy.context.scene.unit_settings.system_rotation = 'RADIANS'
```

```
        # Rename the sphere for easy identification
        sphere.name = f"Implant_{unn}"

        # Optionally, add a material to the sphere for better visualization
        mat = bpy.data.materials.new(name=f"Material_ToothIndex_{unn}")
        mat.diffuse_color = (1, 0, 0, 1)  # Red color
        sphere.data.materials.append(mat)

        # Link the sphere to the collection
        collection.objects.link(sphere)

# Example usag
directory_path = r"G:\My Drive\Research data\TruAbutment Horizontal\Bars\Bars Trios"
process_xml_files(directory_path)
```

Truness

```
import bpy
import csv
import math
import numpy as np
import datetime

print('------------------------- //////////////// ------------------------- ')
```

```
# Goal: compare ds_SB1_01 with prim_SB1_01, prim_SB2_01, omni_SB1_01, omni_SB2_01

def getname(scanner, SB, model, site):
    name = scanner + '_' + SB + '_' + str(model) + '_' + str(site)
    # print('Get name =', name)
    return (name)


def length(a):
    len = math.sqrt(a[0] ** 2 + a[1] ** 2 + a[2] ** 2)
    return len


def cordi_obj(objname):
    objname = objname
    cordinate = np.array([bpy.data.objects[objname].location])
    return cordinate


def cal3Ddev(sitename):
    site_parts = sitename.split("_")
    site_parts[0] = 'E4'
    site_parts[1] = 'ANMA'
    site_parts[2] = '1'
    referencename = "_".join(site_parts)
    controlname = referencename
```

```
    control = np.array(bpy.data.objects[controlname + '_p'].location)
    test = np.array(bpy.data.objects[sitename + '_p'].location)
    dev = np.linalg.norm(control - test)
    print('222 The deviation between ', sitename + '_p', 'and', controlname + '_p', 'is', dev)
    return dev


def dev_ang(name):  # calculate angular deviation of a placed implant
    impname = name
    # print('Calculating angle deviation of', name)
    imp_api_name = impname + '_p'
    imp_coro_name = impname + '_c'

    site_parts = name.split("_")
    site_parts[0] = 'E4'
    site_parts[1] = 'ANMA'
    site_parts[2] = '1'
    referencename = "_".join(site_parts)

    imp00_api_name = referencename + '_p'
    imp00_coro_name = referencename + '_c'

    vector_1 = cordi_obj(imp_api_name) - cordi_obj(imp_coro_name)
    vector_2 = cordi_obj(imp00_api_name) - cordi_obj(imp00_coro_name)
```

```
    unit_vector_1 = vector_1 / np.linalg.norm(vector_1)

    unit_vector_2 = vector_2 / np.linalg.norm(vector_2)

    # print(type(unit_vector_1), unit_vector_2)

    a1 = np.squeeze(np.asarray(unit_vector_1))

    b1 = np.squeeze(np.asarray(unit_vector_2))

    dot_product = np.dot(a1, b1)

    angle = np.arccos(dot_product)

    angle360 = math.degrees(angle)

    print('The angular deviation between ', impname, 'and', referencename, 'is', angle360)


    return angle360




# write data into a CVS file  /Users/Jaden/Desktop/Guided implant placement/Priscilla
Scanbody Project/Measure results

with open(r'/Users/borellaps/Desktop/TRUENESS'+ '.csv',

        'w', newline='') as csvfile:

    fieldnames = ['scanner', 'scanbody', 'repeat', 'Site', '3D_dev_trueness',
'Angular_dev_trueness']

    writer = csv.DictWriter(csvfile, fieldnames=fieldnames)

    writer.writeheader()


    scannerlist = ['E4', 'MEDIT', 'PLANMECA', 'PRIMESCAN', 'TRIOS3', 'TRIOS4', 'TRIOS5',
'MEDITi900']

    scanbodylist = ['ADMA', 'ANMA', 'CYLINDER']

    repeatlist = ['1', '2', '3', '4', '5', '6', '7', '8', '9', '10']

    sitelist = ['3', '5', '7', '10', '12', '14']
```

```
count = 0

for scanner in scannerlist:
    for scanbody in scanbodylist:
        for repeat in repeatlist:
            for site in sitelist:
                objname = getname(scanner, scanbody, repeat, site)

                print('Start ', objname)

                try:
                    dev_3d = cal3Ddev(objname) * 1000

                except Exception as error:
                    dev_3d = 'Exception'
                    print('''////////////// ERROR //////////////''')
                    print(error)

                try:
                    dev_angle = dev_ang(objname)



                except Exception as error:
                    dev_angle = 'Exception'
                    print('''////////////// ERROR //////////////''')
```

```
        print(error)


      writer.writerow(
        {'scanner': scanner, 'scanbody': scanbody, 'repeat': repeat, 'Site': site,
'3D_dev_trueness': dev_3d,
         'Angular_dev_trueness': dev_angle})
      print('write', scanner, scanbody, repeat, site)


      print('----------- Finished measurement on', objname, '-------------')


      count += 1
      print('Completed ', count, ' measurements', '""')
```

Precision

```
import bpy
import csv
import math
import numpy as np
from itertools import combinations


print('------------------------- //////////////// ------------------------- ')




def replace_third_element(s,t):
```

```
    # Split the string by '_'
    parts = s.split('_')

    # Check if there are at least 3 elements
    if len(parts) >= 3:
        # Replace the third element
        parts[2] = t

    # Join the parts back together using '_'
    return '_'.join(parts)

# Goal: compare ds_SB1_01 with prim_SB1_01, prim_SB2_01, omni_SB1_01, omni_SB2_01

def getname(scanner, SB, model, site):
    name = scanner + '_' + SB + '_' + str(model) + '_' + str(site)
    # print('Get name =', name)
    return (name)


def length(a):
    len = math.sqrt(a[0] ** 2 + a[1] ** 2 + a[2] ** 2)
    return len


def cordi_obj(objname):
    objname = objname
```

```
    cordinate = np.array([bpy.data.objects[objname].location])
    return cordinate



def cal3Ddev(a, b):
    point1 = bpy.data.objects[a + '_p'].location
    point2 = bpy.data.objects[b + '_p'].location
    distance_v = point1 - point2
    distance = math.sqrt(distance_v[0] * distance_v[0] + distance_v[1] * distance_v[1] +
distance_v[2] * distance_v[2])

    print(distance_v[0], distance_v[1], distance_v[2])
    print('The distance between ', a, ' and ', b, ' is ', distance)
    return distance




def dev_ang(impname1, impname2):  # calculate angular deviation of a placed implant
    imp_api_name = impname1 + '_p'
    imp_coro_name = impname1 + '_a'

    imp00_api_name = impname2 + '_p'
    imp00_coro_name = impname2 + '_a'

    vector_1 = cordi_obj(imp_api_name) - cordi_obj(imp_coro_name)
    vector_2 = cordi_obj(imp00_api_name) - cordi_obj(imp00_coro_name)
```

```
    unit_vector_1 = vector_1 / np.linalg.norm(vector_1)
    unit_vector_2 = vector_2 / np.linalg.norm(vector_2)
    # print(type(unit_vector_1), unit_vector_2)
    a1 = np.squeeze(np.asarray(unit_vector_1))
    b1 = np.squeeze(np.asarray(unit_vector_2))
    dot_product = np.dot(a1, b1)
    angle = np.arccos(dot_product)
    angle360 = math.degrees(angle)
    print('The angular deviation between ', impname1, 'and', impname2, 'is', angle360)

    return angle360



# ////////////////
scannerlist = ['E4', 'MEDIT', 'PLANMECA', 'PRIMESCAN', 'TRIOS3', 'TRIOS4', 'TRIOS5', 'MEDITi900']
scanbodylist = ['ADMA', 'ANMA', 'CYLINDER']
repeatlist = ['1', '2', '3', '4', '5', '6', '7', '8', '9', '10']
sitelist = ['3', '5', '7', '10', '12', '14']

comparelist = []
combilist = list(combinations(repeatlist, 2))



# write data into a CVS file  /Users/Jaden/Desktop/Guided implant placement/Priscilla Scanbody Project/Measure results
```

```
with open(
      r'/Users/borellaps/Desktop/Precision' + '.csv',
      'w', newline='') as csvfile:
   fieldnames = ['scanner', 'scanbody', 'compare', 'Site', '3D_dev_precision',
'Angular_dev_precision']
   writer = csv.DictWriter(csvfile, fieldnames=fieldnames)
   writer.writeheader()


   count = 0


   for scanner in scannerlist:
      for scanbody in scanbodylist:
         for site in sitelist:
            objname = scanner + '_' + scanbody + '_' + 'tobereplace' + '_' + site
            for comb in combilist:
               comb_names = []


               objname1 = replace_third_element(objname, comb[0])
               objname2 = replace_third_element(objname, comb[1])


               comb_names.append(objname1)
               comb_names.append(objname2)


               print('''


                     Now measuring pricision on:''', comb_names)
```

```
            try:
                dev_3d = cal3Ddev(comb_names[0], comb_names[1]) * 1000
            except Exception as error:
                dev_3d = 'Exception'
                print(error)

            try:
                dev_angle = dev_ang(comb_names[0], comb_names[1])
            except Exception as error:
                dev_angle = 'exception'
                print('''////////////// ERROR //////////////''')
                print(error)

            writer.writerow(
                {'scanner': scanner, 'scanbody': scanbody, 'compare': comb, 'Site': site,
'3D_dev_precision': dev_3d,
                 'Angular_dev_precision': dev_angle})
            print('write', scanner, scanbody, comb, site)

            print('----------- Finished measurement on', objname, '-------------')

            count += 1
            print('Completed ', count, ' measurements', '''


''')
```
